# Data stream dataset of SARS-CoV-2 genome

**DOI:** 10.1016/j.dib.2020.105829

**Published:** 2020-06-10

**Authors:** Raquel de M. Barbosa, Marcelo A.C. Fernandes

**Affiliations:** a Laboratory of Drug Development, Department of Pharmacy, Federal University of Rio Grande do Norte, Natal, RN59078-970, Brazil; b Laboratory of Machine Learning and Intelligent Instrumentation, IMD/nPITI, Federal University of Rio Grande do Norte, Natal 59078-970, Brazil; c Department of Computer Engineering and Automation, Federal University of Rio Grande do Norte, Natal, RN 59078-970, Brazil; d MIT Department of Chemical Engineering, Massachusetts Institute of Technology, Cambridge, MA, 02142, USA; e John A. Paulson School of Engineering and Applied Sciences, Harvard University, Cambridge, MA 02138, USA

**Keywords:** SARS-CoV-2, Data stream, COVID-19

## Abstract

As of May 25, 2020, the novel coronavirus disease (called COVID-19) spread to more than 185 countries/regions with more than 348,000 deaths and more than 5,550,000 confirmed cases. In the bioinformatics area, one of the crucial points is the analysis of the virus nucleotide sequences using approaches such as data stream techniques and algorithms. However, to make feasible this approach, it is necessary to transform the nucleotide sequences string to numerical stream representation. Thus, the dataset provides four kinds of data stream representation (DSR) of SARS-CoV-2 virus nucleotide sequences. The dataset provides the DSR of 1557 instances of SARS-CoV-2 virus, 11540 other instances of other viruses from the Virus-Host DB dataset, and three instances of Riboviria viruses from NCBI (Betacoronavirus RaTG13, bat-SL-CoVZC45, and bat-SL-CoVZXC21).

**Table:** 

Specifications Table	
Subject	Biochemistry, Genetics and Molecular Biology (General)
Specific subject area	Bioinformatics
Type of data	Table
	Number
How data were acquired	NCBI - Genbank - SARS-CoV2 https://www.ncbi.nlm.nih.gov/genbank/sars-cov-2-seqs
	Virus-Host-DB https://www.genome.jp/virushostdb
	Matlab Software
	Excel Software
Data format	Raw and analyzed data are in Matlab file (.mat) and Microsoft Excel file (.xlsx).
Parameters for data collection	The entire dataset was generated using MATLAB 2019b on Windows operating system with Intel Core - i5 6500T 2.5 GHz quad-core processor with 16GB of RAM.
Description of data collection	The raw data were downloaded from NCBI - Genbank, and Virus-Host-DB. The data stream values were generated using Matlab.
Data source location	Laboratory of Machine Learning and Intelligent Instrumentation, IMD/nPITI, Federal University of Rio Grande do Norte.
Data accessibility	https://data.mendeley.com/datasets/g5ktw4y4pz/2

## Value of the Data

• These data are useful because they provide numeric representation of the COVID-2019 epidemic virus (SARS-CoV-2). With this, it is possible to use data stream algorithms.
• All researchers in bioinformatics, computing science, and computing engineering disciplines can benefit from these data because by using this numeric representation, they can apply several stream algorithms and techniques such as TEDA (Typicality and Eccentricity Data Analytic), TEDA-Cloud, TEDA-Cluster and Teda-Class in genomic information.
• Data experiments that use analytic stream techniques in SARS-CoV-2 virus genomic information can be used with this dataset.
• These data represent an simple way to evaluate the SARS-CoV-2 virus genome with stream algorithms.
• Differently of the conventional bioinformatics techniques in which are based on dynamic programming (such as BLAST and other), this approach allows the utilization of different techniques (techniques commons in other areas) to find similarities between genome sequences.

## 1 Data Description

This work presents a dataset of data stream representation (DSR) of SARS-CoV-2 virus nucleotide sequences. The dataset contains two kinds of data, the raw data, and the processing data. The raw data is composed of the 1557 instances of the SARS-CoV-2 virus genome collected from the National Center for Biotechnology Information (NCBI) [1], 11540 instances of other viruses from the Virus-Host DB [2], [3], and the other three specific viruses also collected from NCBI (Betacoronavirus RaTG13, bat-SL-CoVZC45, and bat-SL-CoVZXC21). The last specific three viruses have high similarity with SARS-CoV-2 [4], [5]. The processing data is composed of four kinds of DSR called Direct Mapping (DM), DM with Chaos Game Representation (DM-CGR), *k*-mers mapping (kMersM) and *k*-mers mapping with CGR (kMersM-CGR). *k*-mers is a frequency count metric used in Bioinformatics. Other *k*-mers datasets are presented in [6], [7], [8].

In the Chaos Game Representation (CGR) [8], the genome sequence is transformed in a bi-dimensional signal (1D vector), and after that, this signal passes to infinite impulse response (IIR) filter [9]. The result of CGR is a signal that expressed the density of the bases and, at the same time, the transition between bases because the IIR is a memory system. CGR can be used with the signature of the genome sequence. With *k*-mers representation [10], the genome can be transformed into a 1D or 2D vector that represents the occurrence number of each base (frequency of the bases). *k*-mers also can be used with a signature of the genome sequence. However, in this manuscript, the genome sequence is transformed into a linear stream data, and this type of transformation can be used with stream algorithms. Another important aspect of this dataset is associated with applied CGR not in all sequences but just in each k bases (with mers or not). This strategy maintains the statistical characteristics and reduces the size of the stream.

The data is organized into three main directories: “SARS-CoV-2 data”, “Virus-Host DB data” and “Other viruses data”. Each main directory contains three files called “RawDataTable.mat”, “RawData.mat” and “RawData.xlsx”, and four sub-directories named “DirectMapping”, “DirectMappingCGR”, “kmersMapping” and “kmersMappingCGR”. “RawDataTable.mat”, “RawData.mat” and “RawData.xlsx” files store the raw data information from viruses database; they have the same information, however in the “RawDataTable.mat” the attributes are stored in Matlab table format (after 2013b version), in the “RawData.mat” the attributes are stored in Matlab cell arrays format, and in the “RawData.xlsx” the attributes are stored in a Microsoft Excel file. In the sub-directories “DirectMapping”, “DirectMappingCGR”, “kmersMapping” and “kmersMappingCGR” are stored the DM, DM-CGR, kMersM and kMersM-CGR data stream representation, respectively. Inside each sub-directory the files are called:

• For DM, the DSR was generated for k=1…5 and the files are called “PointsData_1_k=***k***.mat”;
• For DM-CGR, the DSR was generated for k=1…7 and the files are called “PointsDataCGR_1_k=***k***.mat”;
• For kMersM, the DSR was generated for k=2…5 and the files are called “PointsDatakmers_1_k=***k***.mat”;
• For kMersM-CGR:
  • In the directories “Other viruses data” and “SARS-CoV-2 data”, the DSR was generated for k=2…7 and the files are called “PointsDatakmersCGR_1_k=***k***.mat”;
  • In the “Virus-Host DB data”, the DSR was generated for k=2,3,5,and7 and the files are called “PointsDatakmersCGR_1_k=***k***.mat”;

For the main directory “Virus-Host DB data”, the values are stored in 10 files where each *i*-th file is called “PointsData_***k***_k=***k***.mat” for sub-directory “DirectMapping”, “PointsDataCGR_***i***_k=***k***.mat” for DM-CGR, “PointsDatakmers_***i***_k=***k***.mat” for kMersM and “PointsDatakmersCGR_***i***_k=***k***.mat” for kMersM-CGR.

## 2 Experimental design, materials, and methods

The streams were based in nucleotide sequence, **s**, expressed as

(1) $$s=\left[s_{1},\ldots ,s_{n},\ldots ,s_{N}\right]$$

where *N* is the length of sequence and *s_n_* is the *n*th nucleotide of the sequence.

For DM and DM-CGR, the nucleotide sequence, **s**, are grouped in sub-sequences of the *k* bases. The group of sub-sequences can be expressed as

(2) $$B=\left[\begin{array}{c} b_{1}\\ \vdots \\ b_{i}\\ \vdots \\ b_{K} \end{array}\right]=\left[\begin{array}{ccc} s_{1} & \cdots & s_{k}\\ \vdots & \ddots & \vdots \\ s_{k(i-1)+1} & \cdots & s_{k(i-1)+k}\\ \vdots & \ddots & \vdots \\ s_{K-k+1} & \cdots & s_{K} \end{array}\right]$$

where

(3) $$K=k\times \left\lfloor \frac{N}{k}\right\rfloor$$

and the *i*-th vector **b**_*i*_ is a *i*-th group of the *k* nucleotides, that is

(4) $$b_{i}=\left[b_{i,1},\ldots ,b_{i,j},\ldots ,b_{i,k}\right]=\left[s_{k(i-1)+1},\ldots ,s_{k(i-1)+j},\ldots ,s_{k(i-1)+k}\right].$$

For DM, the group of sup-sequences, stored in matrix **B**, are transformed in a sequence of the integer values expressed as

(5) $$c=\left[c_{1},\ldots ,c_{i},\ldots ,c_{K}\right]$$

where **c** is the DM stream stored in dataset. The DM stream, **c**, calculus can be expressed as

(6) $${\left[\begin{array}{c} c_{1}\\ \vdots \\ c_{i}\\ \vdots \\ c_{K} \end{array}\right]}^{T}=f_{\text{map}}\left(B\right)=\left[\begin{array}{c} f_{\text{map}}\left(b_{1}\right)\\ \vdots \\ f_{\text{map}}\left(b_{i}\right)\\ \vdots \\ f_{\text{map}}\left(b_{K}\right) \end{array}\right]$$

where *f*_map_( · ) is the mapping function expressed by

(7) $$c_{i}=f_{\text{map}}\left(b_{i}\right)=\left(\sum_{j=0}^{k-1}4^{j}\times \left(u_{i,j}-1\right)\right)+1$$

and

(8) $$u_{i,j}=\left\{ \begin{array}{cc} 1 & \;\text{for}\;b_{i,j+1}=\text{T}\;\text{or}\;\text{U}\\ 2 & \;\text{for}\;b_{i,j+1}=\text{C}\\ 3 & \;\text{for}\;b_{i,j+1}=\text{A}\\ 4 & \;\text{for}\;b_{i,j+1}=\text{G} \end{array}\right..$$

For DM-CGR, the stream is characterized by vector **a** expressed as

(9) $$a=\left[a_{1},\ldots ,a_{i},\ldots ,a_{K}\right]$$

where the *a_i_* is the *i*-th value of CGR. In CGR (see [11], [12]) each element *a_i_* is a bi-dimensional value expressed as

(10) $$a_{i}=\left(a_{i}^{x},a_{i}^{y}\right)$$

where $a_{i}^{x}$ and $a_{i}^{y}$ are the x-axes and y-axes in bi-dimensional space, receptively. The values of the CGR are calculate using the functions $f_{\text{CGR}}^{x}\left(\cdot \right)$ and $f_{\text{CGR}}^{y}\left(\cdot \right)$ in Matrix **B**, that is

(11) $${\left[\begin{array}{c} \left(a_{1}^{x},a_{1}^{y}\right)\\ \vdots \\ \left(a_{i}^{x},a_{i}^{y}\right)\\ \vdots \\ \left(a_{K}^{x},a_{K}^{y}\right) \end{array}\right]}^{T}=\left(f_{\text{CGR}}^{x}\left(B\right),f_{\text{CGR}}^{y}\left(B\right)\right)=\left[\begin{array}{c} \left(f_{\text{CGR}}^{x}\left(b_{1}\right),f_{\text{CGR}}^{y}\left(b_{1}\right)\right)\\ \vdots \\ \left(f_{\text{CGR}}^{x}\left(b_{i}\right),f_{\text{CGR}}^{y}\left(b_{i}\right)\right)\\ \vdots \\ \left(f_{\text{CGR}}^{x}\left(b_{K}\right),f_{\text{CGR}}^{y}\left(b_{K}\right)\right) \end{array}\right].$$

The function $f_{\text{CGR}}^{x}\left(\cdot \right)$ calculates the x-axes value of the CGR and it can be expressed as

(12) $$a_{i}^{x}=f_{\text{CGR}}^{x}\left(b_{i}\right)=p_{i,k}^{x}$$

where

(13) $$p_{i,j}^{x}=\frac{1}{2}u_{i,j}^{x}+\frac{1}{2}p_{i,j-1}^{x}\text{,}\;\text{for}\;j=1,\ldots ,k$$

and

(14) $$u_{i,j}^{x}=\left\{ \begin{array}{cc} 1 & \;\text{for}\;b_{i,j}=\text{A}\\ -1 & \;\text{for}\;b_{i,j}=\text{T}\;\text{or}\;\text{U}\\ -1 & \;\text{for}\;b_{i,j}=\text{C}\\ 1 & \;\text{for}\;b_{i,j}=\text{G} \end{array}\right..$$

For y-axes, the function, $f_{\text{CGR}}^{y}\left(\cdot \right),$ can be expressed as

(15) $$a_{i}^{y}=f_{\text{CGR}}^{y}\left(b_{i}\right)=p_{i,k}^{y}$$

where

(16) $$p_{i,j}^{y}=\frac{1}{2}u_{i,j}^{y}+\frac{1}{2}p_{i,j-1}^{y}\text{,}\;\text{for}\;j=1,\ldots ,k$$

and

(17) $$u_{i,j}^{y}=\left\{ \begin{array}{cc} 1 & \;\text{for}\;b_{i,j}=\text{A}\\ 1 & \;\text{for}\;b_{i,j}=\text{T}\;\text{or}\;\text{U}\\ -1 & \;\text{for}\;b_{i,j}=\text{C}\\ -1 & \;\text{for}\;b_{i,j}=\text{G} \end{array}\right..$$

For the initial condition, j=0, $p_{i,0}^{x}=\alpha_{x}$ and $p_{i,0}^{y}=\alpha_{y}$ [11], [12]. The dataset was generated with $\alpha_{x}=0$ and $\alpha_{y}=0$.

For kMersM and kMersM-CGR, the nucleotide sequence, **s**, are grouped in *k*-mers sub-sequences [13], [14] in the matrix **H** that can expressed as

(18) $$H=\left[\begin{array}{c} h_{1}\\ h_{2}\\ \vdots \\ h_{i}\\ \vdots \\ h_{N-k}\\ h_{N-k+1} \end{array}\right]=\left[\begin{array}{ccc} s_{1} & \cdots & s_{k}\\ s_{2} & \cdots & s_{k+1}\\ \vdots & \ddots & \vdots \\ s_{i} & \cdots & s_{i+k}\\ \vdots & \ddots & \vdots \\ s_{N-k} & \cdots & s_{N-1}\\ s_{N-k+1} & \cdots & s_{N} \end{array}\right].$$

The kMersM, stream is characterized as a sequence of the integer values expressed as

(19) $$r=\left[r_{1},\ldots ,r_{i},\ldots ,r_{N-k+1}\right]$$

where

(20) $${\left[\begin{array}{c} r_{1}\\ \vdots \\ r_{i}\\ \vdots \\ h_{N-k+1} \end{array}\right]}^{T}=f_{\text{map}}\left(H\right)=\left[\begin{array}{c} f_{\text{map}}\left(h_{1}\right)\\ \vdots \\ f_{\text{map}}\left(h_{i}\right)\\ \vdots \\ f_{\text{map}}\left(h_{N-k+1}\right) \end{array}\right].$$

The function *f*_map_( · ) is the mapping processing characterized by Eqs. (7) and (8). The kMersM-CGR is stored in the vector **z** expressed as

(21) $$z=\left[z_{1},\ldots ,z_{i},\ldots ,z_{N-k+1}\right]$$

where the *z_i_* is the *i*-th value of CGR. Each *i*th element *z_i_* is a bi-dimensional value expressed as

(22) $$z_{i}=\left(z_{i}^{x},z_{i}^{y}\right)$$

where $z_{i}^{x}$ and $z_{i}^{y}$ are the x-axes and y-axes in bi-dimensional space, receptively. The values of the CGR are calculate using the functions $f_{\text{CGR}}^{x}\left(\cdot \right)$ (see Eqs. (12)–(14)) and $f_{\text{CGR}}^{y}\left(\cdot \right)$ (see Equation see Eqs. (15)–(17)) in Matrix **H**, that is

(23) $$\begin{array}{cc} {\left[\begin{array}{c} \left(z_{1}^{x},z_{1}^{y}\right)\\ \vdots \\ \left(z_{i}^{x},z_{i}^{y}\right)\\ \vdots \\ \left(z_{N-k+1}^{x},z_{N-k+1}^{y}\right) \end{array}\right]}^{T} & =\left(f_{\text{CGR}}^{x}\left(H\right),f_{\text{CGR}}^{y}\left(H\right)\right)\\ & =\left[\begin{array}{c} \left(f_{\text{CGR}}^{x}\left(h_{1}\right),f_{\text{CGR}}^{y}\left(h_{1}\right)\right)\\ \vdots \\ \left(f_{\text{CGR}}^{x}\left(h_{i}\right),f_{\text{CGR}}^{y}\left(h_{i}\right)\right)\\ \vdots \\ \left(f_{\text{CGR}}^{x}\left(h_{N-k+1}\right),f_{\text{CGR}}^{y}\left(h_{N-k+1}\right)\right) \end{array}\right]. \end{array}$$

**Fig. 1:**
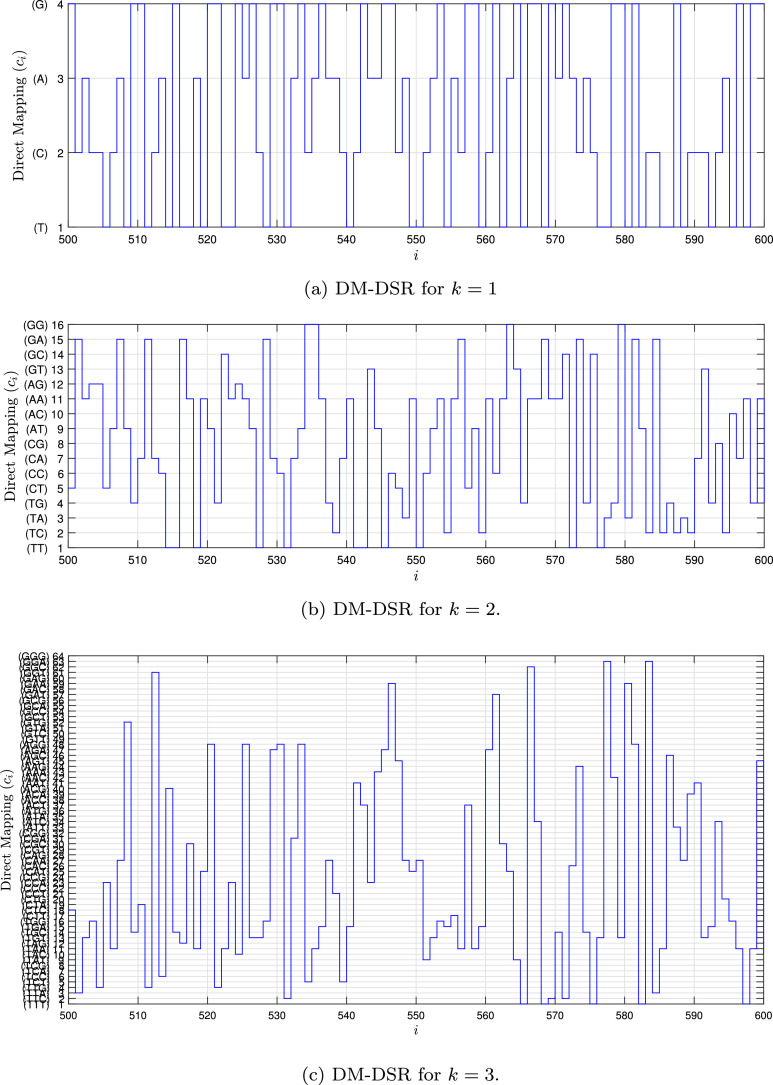
Example of the DM-DSR values for the SARS-CoV-2 sequence (i=500…600) stored in dataset (MT126808 - Brazil).

**Fig. 2:**
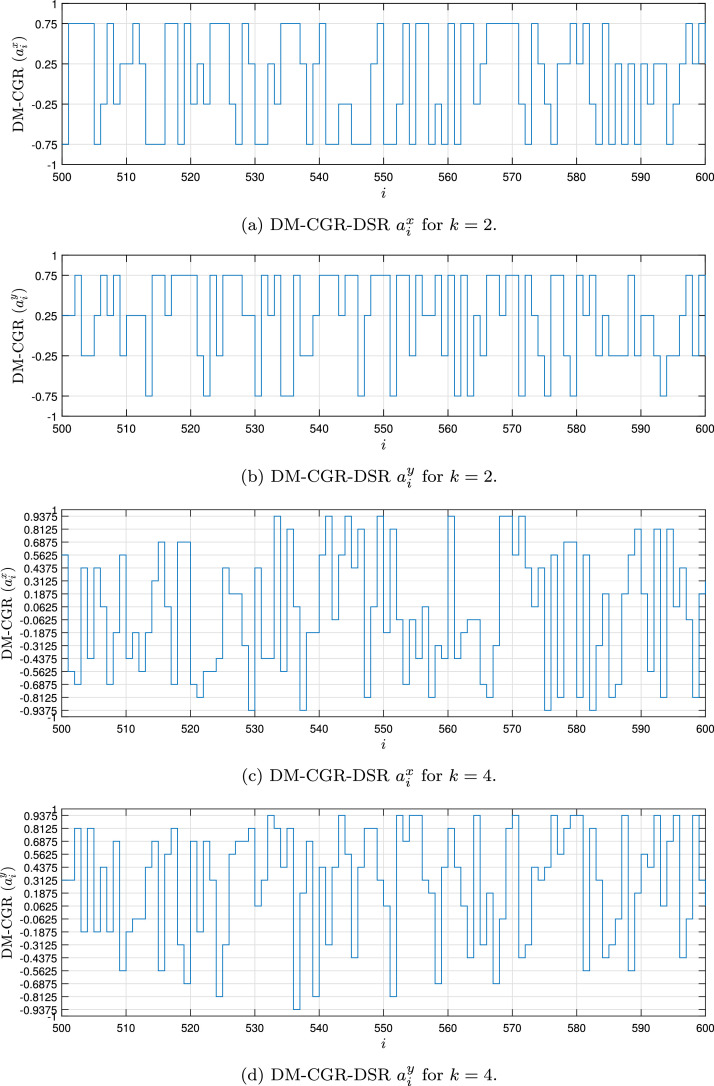
Example of the DM-CGR-DSR values for the SARS-CoV-2 sequence (i=500…600) stored in dataset (MT126808 - Brazil).

**Fig. 3:**
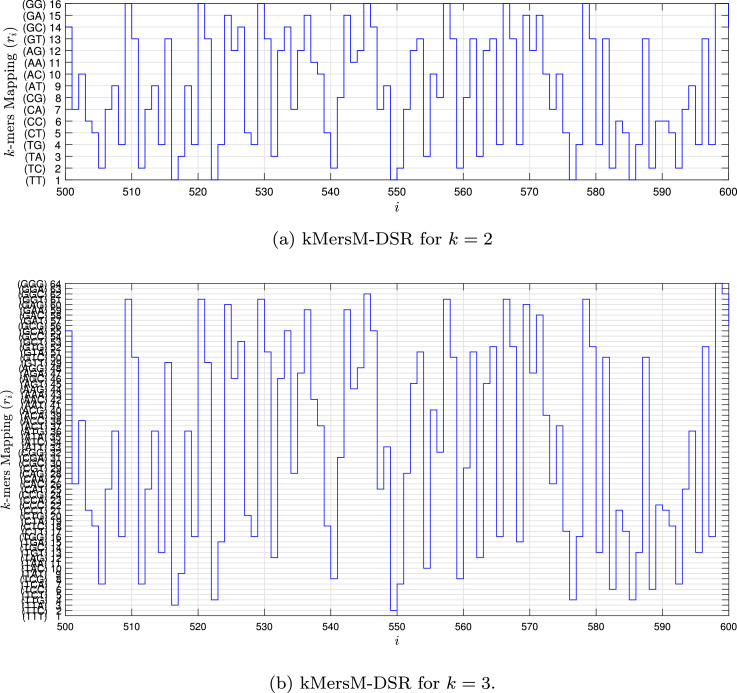
Example of the kMersM-DSR values for the SARS-CoV-2 sequence (i=500…600) stored in dataset (MT126808 - Brazil).

**Fig. 4:**
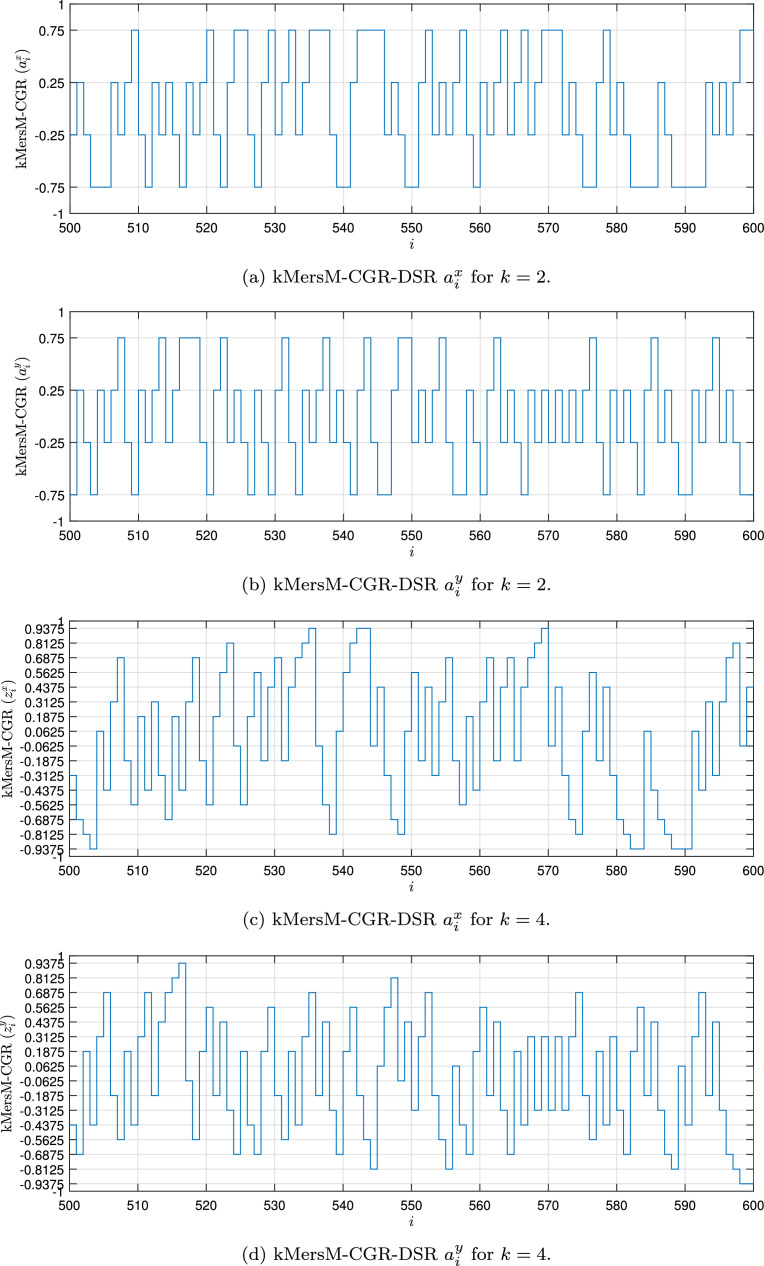
Example of the kMersM-CGR-DSR values for the SARS-CoV-2 sequence (i=500…600) stored in dataset (MT126808 - Brazil).

Fig. 1, Fig. 2, Fig. 3, Fig. 4 show the DSR examples for SARS-CoV-2 from Brazil, respectively.

## Declaration of Competing Interest

The authors declare that they have no known competing financial interests or personal relationships that could have appeared to influence the work reported in this paper.

## Appendix A Supplementary materials

Supplementary Data S1 Supplementary Raw Research Data. This is open data under the CC BY license http://creativecommons.org/licenses/by/4.0/ Supplementary Data S1
